# Breakdown of COVID effects on students’ mental health at the beginning of the pandemic

**DOI:** 10.1371/journal.pmen.0000363

**Published:** 2025-06-26

**Authors:** Elizabeth Amona, Alexis West, Afton White, Indranil Sahoo, David M. Chan, Punit Gandhi, Yanjun Qian

**Affiliations:** 1 Department of Statistical Sciences and Operations Research, Virginia Commonwealth University, Richmond, Virginia, United States of America; 2 Department of Biomedical Engineering, Virginia Commonwealth University, Richmond, Virginia, United States of America; 3 Department of Mathematics and Applied Mathematics, Virginia Commonwealth University, Richmond, Virginia, United States of America; Universidad de Huelva Facultad de Educacion, SPAIN

## Abstract

Students at a large mid-Atlantic university were surveyed during the early months of the COVID-19 pandemic to assess its impact on their mental health. This study implements a combined analysis of categorical and text-based survey data using chi-squared tests and correspondence analysis to examine subgroup differences in the mental health impacts of the pandemic. When examining different subgroups, it was observed that first-generation students, who often cited stress and lack of motivation, reported being less affected by the pandemic compared to non-first generation students. The latter group more frequently mentioned experiencing depression, anxiety, isolation, challenges at home, lack of routine, and other mental health concerns. Additionally, female students appeared to be more impacted than male students, with female respondents highlighting issues of depression, lack of motivation and routines, anxiety, and other home-related mental health struggles. While other student subgroups were similarly affected, differences emerged in the specific ways they were impacted. STEM students were more likely to cite challenges with home life, feelings of isolation, and negative emotions, whereas non-STEM students more frequently reported depression, anxiety, and stress. Graduate students tended to describe the effects of COVID-19 in terms of depression, stress, and disrupted routines, while undergraduates were more likely to mention isolation, negative emotions, and challenges at home. Finally, age-based differences were also evident: younger students (18–20 years old) often cited home-related challenges, students aged 21–24 mentioned anxiety, lack of routine, and other mental health struggles, and the oldest group (25+ years) most frequently referenced stress and depression as consequences of the pandemic.

## Introduction

The COVID-19 pandemic disrupted daily routines and social interactions, creating widespread challenges that touched nearly every aspect of life. As the world faced an unprecedented health crisis, its profound impact on mental health became increasingly evident [1, 2]. For many, routines were upended, social connections severed, and a profound sense of uncertainty loomed, altering the way people lived, worked, and connected with one another. What began as a public health emergency quickly evolved into a psychological crisis, affecting individuals from all walks of life. Feelings of fear, grief, and isolation were pervasive, creating fertile ground for anxiety, depression, and other mental health conditions. Reports of sleep disturbances, overwhelming stress, and difficulty concentrating became common as individuals struggled to adapt to new realities. The strain was compounded by financial instability, job losses, and the grief of losing loved ones, intensifying the challenges faced by individuals across the globe. The widespread mental health impact was captured in systematic reviews and meta-analyses, which highlighted that nearly one in three individuals experienced significant levels of stress, anxiety, or depression during the pandemic [3, 4].

The World Health Organization (WHO) reported a 25% global increase in anxiety and depression during the first year of the pandemic, reflecting the far-reaching consequences of this health crisis [4]. Anxiety symptoms were reported in a range of 6.33% to 50.9%, while depression symptoms varied from 14.6% to 48.3% among the general population [5]. These statistics underline the significant psychological burden experienced by diverse demographics during the pandemic.

The role of isolation and loneliness emerged as a critical factor exacerbating mental health challenges. Studies documented that the prevalence of loneliness increased from 30.75% pre-pandemic to 50.5% during the pandemic [6]. This heightened sense of isolation was closely linked to worsening mental health conditions, predicting a threefold increase in emotional distress among those frequently experiencing loneliness [7]. Social distancing measures and restrictions, though essential to curbing the virus’s spread, further amplified feelings of social disconnection and lack of emotional support [8]. The long-term effects of the pandemic on mental health have also been significant. Individuals recovering from COVID-19 reported persistent symptoms of anxiety, post-traumatic stress disorder (PTSD), and poor sleep quality for months following their recovery. A systematic review revealed that fatigue and anxiety disorders continued to affect some individuals up to a year post-recovery, emphasizing the enduring psychological toll of the pandemic [9].

Certain subpopulations bore a disproportionate share of the mental health burden. Women, younger adults, and individuals with pre-existing mental health conditions were particularly vulnerable to anxiety and depression [5, 10]. Economic challenges further deepened these disparities, with individuals experiencing job losses reporting mental health symptoms at rates significantly higher than their employed counterparts. Specifically, 53% of those who lost jobs reported symptoms of anxiety and/or depression compared to 30% among those who retained employment [10]. Young adults were especially affected, with studies indicating a 50% prevalence of anxiety and depression among this group during the pandemic [5]. Stress and psychological distress were widespread across various demographics during the COVID-19 pandemic.

While the mental health burden was significant for the general population, university students emerged as a particularly vulnerable subgroup. This group faced unique challenges worsened by academic pressures, social isolation, and disruptions to their educational experience, making them a focus of growing research efforts. Studies examining the mental health of college students during the pandemic provide deeper insights into the extent and specific nature of these impacts. Son *et al*. [11] conducted interview surveys with 195 students at a large public university in the United States, revealing that 71% reported increased stress and anxiety. Key stressors included fears for personal and family health (91%), difficulty concentrating (89%), disrupted sleep (86%), and decreased social interactions (86%). Similarly, Wang *et al*. [12] surveyed 2,031 undergraduate and graduate students at Texas A&M University and found 48.14% exhibited moderate-to-severe depression, 38.48% reported moderate-to-severe anxiety, and 18.04% experienced suicidal thoughts. Academic concerns, health worries, and daily disruptions emerged as dominant stressors. While studies like [11] and [12] focused on stressors at specific universities, broader surveys provide insights into trends across multiple institutions.

U.S.-based surveys further illustrate the pandemic’s toll on students’ mental health. In May-June of 2020, the Student Experience in the Research University (SERU) Consortium administered a special survey on the impact of COVID-19 at ten public research universities in the U.S. [13]. Chirikov *et al*. [14] analyzed SERU data from over 30,000 undergraduates and 15,000 graduate students across nine universities, finding that 35% of undergraduates and 32% of graduate students screened positive for major depressive disorder, while 39% showed generalized anxiety symptoms. Rates were notably higher among students struggling with remote instruction. Similarly, Copeland *et al*. [15] reported significant increases in externalizing problems and attention issues among 675 first-year students, with wellness programs mitigating some impacts. Kim *et al*. [16] compared survey data before and during the pandemic, revealing sharp increases in depression (Odds Ratio (OR) = 1.32), alcohol use disorder (OR = 1.70), and binge-eating disorder (OR = 1.54). These trends were particularly pronounced among women and Black students, highlighting disparities within the student population.

Kecojevic *et al*. [17] reported high levels of depression, anxiety, and stress among 162 college students, with academic struggles and employment losses as significant contributors. Both [11] and [17] identified academic disruptions and fear of infection as dominant stressors, emphasizing a shared experience among U.S. students. In another survey, Lee *et al*. [18] found that students nearing graduation experienced elevated rates of anxiety (60.8%), loneliness (54.1%), and depression (59.8%), driven by concerns for loved ones and strained family relationships. Meanwhile, Giuntella *et al*. [19] documented significant lifestyle disruptions, including reduced physical activity, increased screen time, and a 90% rise in clinical depression rates compared to pre-pandemic levels.

Browning *et al*. [20] surveyed 2,534 students across seven U.S. universities and identified female gender, poor health, and excessive screen time as key risk factors for elevated psychological distress. Similarly, Chen *et al*. [21] surveyed 1,173 students in the United Kingdom, reporting high rates of depression (53.4%) and anxiety (51.5%). Stressors such as financial struggles and worsened relationships significantly impacted mental health outcomes. Li *et al*. [22] provided a global perspective through a meta-analysis of 27 studies involving over 700,000 students, finding a 39% prevalence of depression and 36% prevalence of anxiety, with rates varying by geographic region and survey timing.

The mental health impact of COVID-19 on students extends beyond general challenges and varies significantly across different demographic groups. Among these, first-generation college students faced unique and disproportionate struggles, particularly related to financial, academic, and emotional stressors. Soria *et al*. [23] used data from the SERU survey to compare the experiences of first-generation and non-first generation students during the pandemic. Their findings revealed that first-generation students were significantly more likely to experience financial hardships, food and housing insecurity, and unsafe living conditions, which contributed to higher rates of mental health disorders among first-generation students compared to their peers. Building on these findings, Soria *et al*. [24] analyzed a subset of over 7,000 responses from the same dataset to explore clinically significant mental health outcomes. The peer-reviewed study found that major depressive disorder and generalized anxiety disorder were disproportionately prevalent among first-generation students.

Raposa *et al*. [25] further explored the mental health experiences of first-generation students through surveys and interviews conducted at three colleges, with 314 survey responses collected during Fall 2019 and Spring 2020, alongside 28 in-depth interviews. Their findings revealed substantial increases in depressive symptoms and perceived stress following the onset of the pandemic, particularly among female first-generation students. The study also found that first-generation students were significantly less confident in seeking mental health support during the pandemic, with a marked decline in mental healthcare usage.

Differences in mental health outcomes between graduate and undergraduate students during the pandemic have also been a significant focus of research. Dial *et al*. [26] directly compared graduate and undergraduate students, surveying approximately 900 undergraduates and 300 graduates across three universities. The results showed that undergraduates reported higher levels of perceived stress, repetitive negative thinking, and reduced positive mood compared to graduate students. Undergraduates also felt less supported by professors, suggesting that the academic and social pressures of the pandemic may have weighed more heavily on this group. However, both graduate and undergraduate students experienced a general increase in mental health difficulties during the pandemic. Villatoro *et al*. [27] surveyed around 800 Texas college students and presented a comparative analysis of mental health impacts between graduate and undergraduate students. The findings indicated that undergraduates reported significantly higher levels of poor mental health related to the pandemic.

Several studies examining the mental health impact of COVID-19 on students have consistently reported significant gender disparities, with female students emerging as a particularly vulnerable group. Browning *et al*. [20] surveyed over 2,500 students across seven U.S. universities and identified female gender as a key risk factor for elevated psychological distress during the pandemic. Women reported significantly higher levels of anxiety, stress, and emotional distress compared to their male counterparts. Similarly, Chen *et al*. [21] found that female students in the United Kingdom exhibited disproportionately higher rates of depression and anxiety, with worsened relationships and financial difficulties acting as major contributing stressors.

Kim *et al*. [16] further supported these findings through a nationwide U.S. survey, showing sharper increases in depression and alcohol use disorder among women compared to men. These trends highlight the compounded pressures female students faced, including academic challenges, health concerns, and social isolation. Fruehwirth *et al*. [28] also observed significant gender disparities, reporting that female first-year students experienced a greater rise in anxiety and depression due to social isolation and difficulties adapting to remote learning environments.

In the age aspect, while the COVID-19 pandemic had a significant impact on the mental health of university students, younger students aged 18–24 experienced disproportionately higher levels of anxiety, loneliness, and depression compared to older peers [18]. These challenges were largely driven by disrupted academic routines and the loss of social connections, which are particularly crucial for students in this age group as they navigate key developmental and transitional phases of their lives.

While many studies examined mental health disparities across various demographics during the COVID-19 pandemic, specific breakdowns comparing STEM and non-STEM students remain limited or under-explored in the literature.

Rich data sets taken within the first couple of months of the pandemic at diverse universities are not common. The analysis of these complex data sets present a variety of problems. These include the size of the data set given diversity of the individuals within a large population, the numerous types of reasons given for the challenges during this unique time in their history, and free from of the responses in order to get a broad perspective from the students.

In this study, we examine the data collected from students at a mid-Atlantic US University during the early months of the COVID-19 pandemic to assess differences in its impact across various subgroup populations. The goal of this research is to better understand if and when there are differences in how subgroups of the population are affected differently by the pandemic by utilizing the collected data. To address the challenges of examining this complex data set, we employed a variety of techniques to obtain useful observations. We used the standard Person’s chi-squared test of independence to check whether frequency data across subgroups exhibited similar patterns. Additionally, we examined qualitative data from short-answer responses that explained the impact of the pandemic on mental health. In order to analyze this data, we employed a correspondence analysis technique to estimate differences in patterns in this data. By employing a statistical methodology that integrates both categorical and text-based survey data, we concluded that some subgroups experienced varying levels of severity or provided different reasons for the pandemic’s impact, while other subgroup comparisons demonstrated differences specifically in the reasons cited for its effects.

## Methods

Data from undergraduate and graduate students at a mid-Atlantic US University was collected between April 9, 2020 through June 3, 2020. Participants were recruited to participate via the university’s daily digital news and events newsletter, as well as via targeted emails to student email lists from the major colleges and schools within the university. Participants received a $20 gift card as compensation for their participation.

All participants were required to be at least 18 years of age and enrolled at the large, mid-Atlantic university. Potential participants were first asked to complete a screening questionnaire to verify their status and all qualified respondents were invited to participate. They were provided a link to complete the online survey using RedCAP (Research Electronic Data Capture, Version 4.14.5). All study procedures were approved by the authors’ Institutional Review Board (Protocol #HM20016878). The sample consisted of a total *N* = 480 students in which all participants provided written consent prior to participation. Descriptive characteristics of the sample can be found in Table 2.

This study focused on the impact of COVID-19 on the mental health of the students. In particular, differences in the impact based on demographic information collected, such as age, major, sex, first-generation status, and graduate/undergraduate status, were examined. Including the demographic information, the specific COVID questions took the form:

1. Please respond with how much you agree/disagree with the following statement: Coronavirus/COVID-19 is affecting my mental health. (0=strongly disagree, 5=neither disagree/agree, 10=strongly agree)
2. Please respond with how much you agree/disagree with the following statement: Coronavirus/COVID-19 is affecting my education. (0=strongly disagree, 5=neither disagree/agree, 10=strongly agree)
3. On a scale of 1-10, how socially isolated do you feel because of the coronavirus/COVID-19? (0=not at all, 5=moderately, 10=extremely )

We will focus on the first question about the COVID-19 impact rating on the mental health of the students.

In addition to these rating scale questions, participants were also asked the following:

1. How is COVID affecting your mental health?
2. How is COVID affecting your education (e.g., learning experiences, grades, etc.)
3. What types of social interactions are you missing most?
4. Do any of the social interactions you are missing surprise you? If so, how?
5. What are you doing to help you feel connected to others during COVID?
6. What resources or supports have been helpful to you during COVID?

From all of these questions, the analysis will be centered on the first question above, relating to the mental health of the participants. Answers to these questions were more frequently answered, and the free response included more detailed information that was used to classify the reasons participants gave for their impact ratings. These results were than analyzed in two different ways: impact between different demographic subgroups, and reasons given for the impact.

### Chi-square test of independence

First, to assess whether there were significant differences in the distribution of COVID-19 impact ratings across various demographic groups, we employed Pearson’s chi-square ($\chi^{2}$) test of independence. This test evaluates the likelihood that any observed differences in distributions occurred by chance, allowing us to determine whether certain demographic characteristics were associated with varying levels of reported impact.

The analysis categorized student ratings into three impact levels: little (0-4), moderate (5-7), and significant (8-10). We then examined these impact distributions across different demographic groups, including sex, field of study, first-generation status, academic status, and age groups. For age, specific comparisons were made between three categories: 18-20 years, 21-24 years, and 25+ years.

The chi-square statistic was calculated using the formula:


$$\chi^{2}=\sum \frac{(O_{i}-E_{i}{)}^{2}}{E_{i}}$$

where *O*_*i*_ represents the observed frequency in each category, and *E*_*i*_ is the expected frequency under the null hypothesis of independence. The degrees of freedom for each test were determined by (r−1)(c−1), where *r* is the number of rows (impact categories) and *c* is the number of columns (demographic groups).

### Correspondence analysis

Secondly, to see if there were differences in the reasons given in how the subgroups were affected, regardless of differences in impact levels, we used Correspondence Analysis (CA) [29], which is a statistical technique used to explore and visually represent relationships among categorical variables in multidimensional space [30]. It works by transforming contingency tables and uncovering patterns that may not be evident through traditional statistical methods [31]. Unlike many statistical techniques, CA does not require strict assumptions about data distribution, which makes it well-suited for analyzing data across various fields, including psychology, sociology, and education [32]. CA has been widely used in different studies to examine complex relationships within categorical data. For example, in educational research, CA has helped reveal connections between students’ perceptions and educational factors, showing how demographic factors interact with students’ academic experiences [33]. In medical research, CA has been valuable for identifying links between mental health conditions and demographic variables, enabling researchers to design more targeted interventions for different population groups [34].

In this study, we apply CA to examine the impact of various mental health factors on university students from different demographic groups during the COVID-19 pandemic. Two researchers collaborated to analyze the free response answers to determine a set of different factors commonly mentioned in the students’ responses as having an impact on mental health across the respondents. Subsequently, they categorized each free response based on whether or not a particular factor was present within the response. Each free response could be assigned to none, one, or multiple of the common factors. To ensure consistency, the two researchers must reach consensus on those questionable categorizations by consulting with other team members. Of the 480 participants in the survey, 75 (15.6%) of participants did not provide a free response answer or provided responses that did include a discernible factor. Table 1 provides the nine factors identified from the students’ free responses, as well as sample responses from each category. The packages FactoMineR [35] and factoextra [36] were used to perform the Correspondence Analysis, with all analyses conducted using R version 4.4.1 [37].

**Table 1:** Factors identified from free responses as impacting mental health. For each factor an example excerpt from a free response is included that was used as a basis for identifying that factor.

Factor affecting mental health	Sample of relevant excerpt from free response
Isolation/Loneliness	“I’m extroverted and the isolation is very taxing."
Depression	“I am deeply depressed from watching the world collapse and feeling so helpless and useless."
Anxiety	“I had general anxiety even before the pandemic, so this has just been adding to it."
Negative Feelings	“I constantly have panic attacks or moments of sadness."
Lack of Motivation/Structure	“The lack of a concrete schedule from not attending classes and extracurricular activities has distorted the passage of time and days feel much longer than they should be."
Stress/Worry	“I feel like I am constantly stressed about me or my family contracting it, and it worries me because I care about my health, but even more about their health."
Misc. Mental Health Issues	“This situation has caused me to struggle even more with my recovery from an eating disorder."
Issues With Home Life/Moving Back Home	“Being away from campus has taken my independence away which has taken a toll on me."
No Effects/Positive Effects	“Giving me time to really focus on myself and develop new hobbies."

#### Mathematical framework of CA.

The first step in Correspondence Analysis (CA) involves constructing a contingency table, where each cell represents the relative frequency (*f*_*ij*_) of occurrences between two or more categorical variables. In this study, for example, we examine the frequency of mental health factors (e.g., anxiety, depression) across various student demographic groups (e.g., STEM vs. non-STEM). The contingency table serves as the basis for identifying associations between categories.

Unlike traditional contingency tables, here participants were categorized to more than one reasons for impact based on their text responses. As a result, row and column totals exceed the sample size, reflecting the frequency of the factors reported rather than the sample size. This distinction is important because it leads to inflated marginal totals compared to standard contingency tables.

The CA process begins with a chi-square analysis as an initial step to assess whether associations between rows and columns deviate from expectations under independence. While we omit detailed chi-square equations, this step evaluates the significance of deviations before proceeding to dimensionality reduction. To extract meaningful dimensions, we compute the relative frequencies (*f*_*ij*_) directly from the data, where each cell represents the proportion of responses within the total:


$$f_{ij}=\frac{n_{ij}}{N}$$

where *n*_*ij*_ is the count of occurrences in cell (i,j), and *N* is the grand total of all responses. The row and column proportions are defined as:


$$f_{i}=\sum_{j}f_{ij}\;\text{and}\;f_{j}=\sum_{i}f_{ij}$$

where *f*_*i*_ represents the row marginal proportion, and *f*_*j*_ represents the column marginal proportion.

Next, standardized residuals are calculated to quantify deviations between observed and expected values:


$$r_{ij}=\frac{f_{ij}-f_{i}\cdot f_{j}}{\sqrt{f_{i}\cdot f_{j}}}$$

These residuals capture variations and form the input for dimensionality reduction [31].

Following the computation of residuals, Singular Value Decomposition (SVD) is applied to the matrix of standardized residuals:


$$R=U\cdot D\cdot V^{T}$$

where *U* represents row profiles, $V^{T}$ represents column profiles, and *D* contains singular values indicating the importance of each dimension [29]. This step allows us to visualize relationships between categories in reduced-dimensional space, making it easier to interpret patterns and associations.

The outputs of CA are typically presented in a reduced-dimensional space, allowing for the visualization of relationships between the categorical variables. In this space, categories that are closer together indicate a stronger association, with the distance between them reflecting the strength of their relationship. The eigenvalues associated with each dimension explain the variance captured by that dimension, where larger eigenvalues indicate that a greater proportion of the total variance is being explained. Eigenvalues also help determine the dimensional importance, guiding the selection of the most meaningful dimensions to retain for interpretation.

Dot plots or biplots provide a graphical representation where both row and column categories are displayed, aiding in the visual interpretation of interactions between variables [30]. These visualizations help reveal patterns and relationships that may not be immediately apparent in the original contingency table. By examining the proximity of points and their orientation relative to the axes, insights can be gained into the structure of the data and the nature of the associations between categories.

## Results

We first examine the impact of COVID on each demographic subgroup. Table 2 gives the breakdown of the severity of how subcategories were affected by the pandemic. We note that each demographic may add up to a different total, due to some individuals not being included in the subgroups presented, for example, a student may not have identified as male or female.

**Table 2:** Frequency impact of COVID-19 of students during the first couple of months of the pandemic. (N = 480)

Category	%-age (n)	COVID-19 Impact Rating		
		Little Impact (0-4)	Moderate Impact (5-7)	Significant Impact (8-10)
**Sex**				
Female	80.21% (n = 385)	45 (11.69%)	158 (41.04%)	182 (47.27%)
Male	14.38% (n = 69)	16 (23.19%)	27 (39.13%)	26 (37.68%)
**Age**				
18 to 20	37.92% (n = 182)	21 (11.54%)	75 (41.21%)	86 (47.25%)
21 to 24	43.96% (n = 211)	25 (11.85%)	98 (46.45%)	88 (41.71%)
25 +	18.13% (n = 87)	16 (18.39%)	28 (32.18%)	43 (49.43%)
**Field of Study**				
STEM	56.46% (n = 271)	40 (14.76%)	120 (44.28%)	111 (40.96%)
Non-STEM	39.38% (n = 189)	21 (11.11%)	72 (38.10%)	96 (50.79%)
**1^*st*^ Generation Status**				
1st generation	19.58% (n = 94)	13 (13.83%)	49 (52.13%)	32 (34.04%)
Non - 1st generation	74.38% (n = 357)	48 (13.45%)	136 (38.10%)	173 (48.46%)
**Academic Status**				
Graduate Student	24.38% (n = 117)	23 (19.66%)	43 (36.75%)	51 (43.59%)
Undergraduate Student	72.29% (n = 347)	39 (11.24%)	150 (43.23%)	158 (45.53%)

Table 3 gives the results of the Pearson’s Chi-squared tests of independence of the frequencies for each subgroups in Table 2. These results determined how likely it is that any observed difference between the frequency of the ratings among the different demographic subsets arose by chance. Based on the chi-square analysis and assuming a p-value of less than 0.05, there were significant differences between male and female students as well as between first-generation students compared with non-first generation students. All others demographic subgroups did not show statistically significant differences in the frequency of the severity of COVID impact. Thus statistically, the other groups were impacted similarly to each other in terms of severity.

**Table 3:** Chi-square analysis of demographic factors and COVID-19 impact ratings.

Demographic Factor	Chi-square Statistic	p-value
**First Generation Status**	** $\chi^{2}\left(2\right)\;=\;6.9823$ **	**0.03047**
**Sex**	** $\chi^{2}\left(2\right)\;=\;6.9867$ **	**0.0304**
STEM vs non-STEM	$\chi^{2}(2)=4.5316$	0.1037
Undergrad vs Graduate	$\chi^{2}(2)=5.5967$	0.06091
Age Group		
Age 18-20 vs. Age 21-24	$\chi^{2}(2)=1.2957$	0.5232
Age 18-20 vs. Age 25+	$\chi^{2}(2)=3.3194$	0.1902
Age 21-24 vs. Age 25+	$\chi^{2}(2)=5.7147$	0.05742

In addition to the frequencies, we also examined the breakdown of the reasons given by the students for the pandemic’s impact to each subgroup. This allowed us to observe whether there were differences in how each subgroup was impacted even in cases where there may not be a difference in the frequencies of the severity of COVID impact between the subgroups. Thus it is possible that the reasons one subgroup was impacted could be very different from how another subgroup was impacted regardless of the results in Table 3. To accomplish this, we used the correspondence analysis (CA), which yielded differences in subgroups. We discuss these results in the following section broken down by demographic subgroups.

### First-generation versus non-first generation students

A *first-generation student* is defined as a student in which neither parent received a college degree. If one or both parents had obtained a college degree, the student was classified as a non-first generation. As previously mentioned, the chi-square analysis showed a difference in the severity of COVID impact between first-generation students and non-first generation students. In particular non-first generation students were more severely impacted by the epidemic than first-generation students.

**Fig 1:**
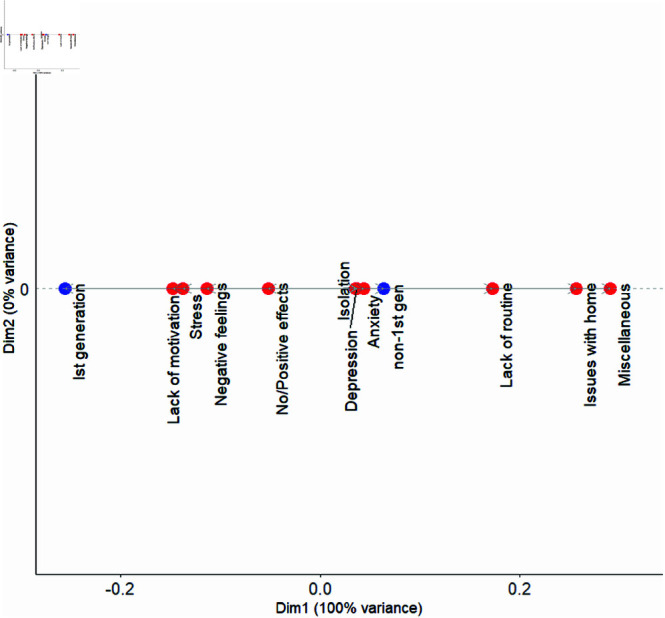
CA dot plot showing the relationship between mental health factors and First-generation vs. Non-First-generation groups. Factors such as stress, lack of motivation, and negative feelings were more associated with the First-generation category, while isolation, depression, anxiety, lack of routine, issues with home, and miscellaneous factors were linked to Non-First-generation students.

To further explore these differences using CA on the reasons given by students (see Table 1) that identified specific mental health-related factors, a CA dot plot was created (see Fig 1). By examining the distance between the dots representing first-generation students and non-first generation students with each factor, we see that first-generation students were more closely linked with factors such as stress and lack of motivation. In contrast, non-first generation students were associated with depression, isolation, anxiety, lack of routine, issues with home, and various miscellaneous challenges, which include trauma, sleep disturbances, self-harm, and eating disorders.

### Female versus male students

The only other subgroups that were found to be impacted differently in terms of impact severity was the female versus male subgroups. As previously mentioned the Pearson’s Chi-squared value indicated a significant association between sex and the level of effect observed with a p-value under 0.05.

**Fig 2:**
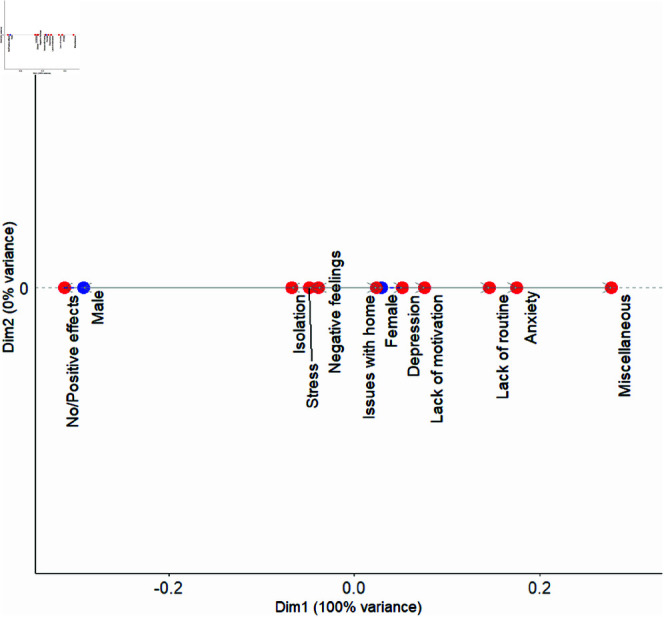
CA dot plot showing the relationship between mental health factors and sex groups. The most prominent factor impacting the male students No/Positive effects. On the other hand, factors such as issues with home, depression, lack of motivation, lack of routine, anxiety, and miscellaneous factors were found to affect the Female students. Isolation, stress and negative feelings had a stronger effect on the Female students compared to their Male counterparts.

In terms of the correspondences analysis shown in Fig 2, we observe that for males more frequently gave a response that was interpreted as No/Positive effects. In contrast, female students are more closely associated with a range of mental health factors, such as depression, lack of motivation, lack of routine, anxiety, miscellaneous factors, and issues with home. This likely due to a few factors. First, there were many more individuals identifying as female that participated in survey than those identifying as male. Secondly, males typically are more reticent in identifying mental health issues compared to their female counterparts [38–41].

### STEM versus non-STEM students

Students majoring in science, technology, engineering, mathematics, or closely related fields are considered STEM students in this study, where all other students are categorized as non-STEM majors. The Chi-squared test did not result in statistical significance between severity impact of the STEM and non-STEM students.

**Fig 3:**
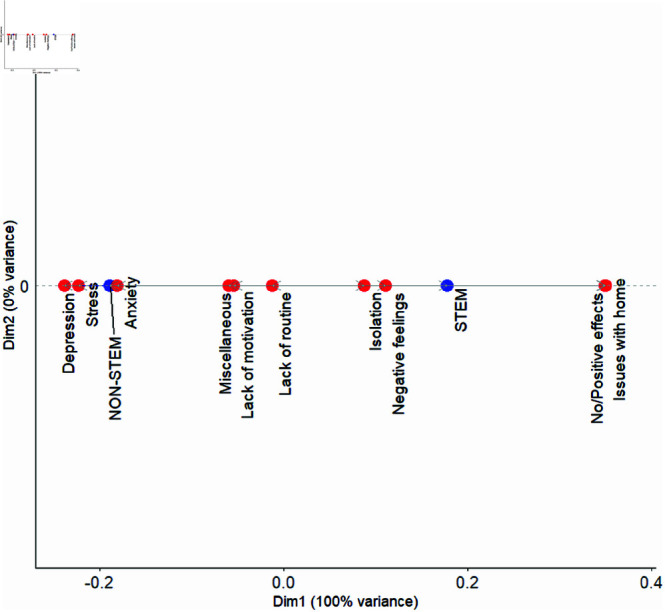
CA dot plot showing the relationship between mental health factors and STEM vs. non-STEM groups. Factors such as depression, stress, and anxiety were associated with the non-STEM category, while factors such as isolation, negative feelings, issues with home, and No/Positive effects were more linked to STEM students. Miscellaneous factors, lack of motivation and lack of routine affected both categories similarly.

The CA dot plot seen in Fig 3 shows that the reasons for COVID impact for non-STEM students more closely aligned with the factors depression, stress, and anxiety. STEM majors, on the other hand, showed closer associations with isolation, negative feelings, issues with home, and No/Positive effects. In this case the factors lack of routine and motivation along with miscellaneous factors were interpreted to affect both categories more or less equally, given they were placed evenly between the subgroups.

### Graduate versus undergraduate students

The chi-squared test involving graduate and undergraduate students obtained a p-value of 0.06091, which suggests there is insufficient evidence to conclude a significant difference in the distribution of impacts between graduate and undergraduate students.

**Fig 4:**
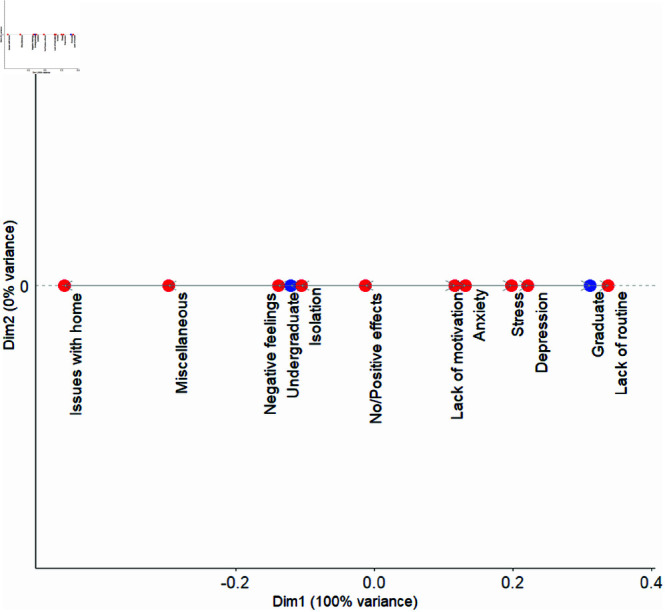
CA dot plot showing the relationship between mental health factors and Graduate vs. Undergraduate students. Factors such as issues with home, miscellaneous factors, negative feelings, and isolation were associated with the Undergraduate students, while factors including lack of motivations, anxiety, stress, depression, and lack of routine structure were linked to Graduate students.

Even though there was no significant difference in the distribution of impact between graduate and undergraduate students, the CA plot (see Fig 4) does reveal differences between mental health factors voiced by these student groups. For undergraduates, the plot shows a closer connection with factors such as issues with home, miscellaneous factors, negative feelings, and isolation. In contrast, graduate students were more closely affected by factors such as stress, depression, and lack of routine. There did not appear to be differences in these groups in relation to lack of motivation or anxiety.

### Age analysis

The pairwise analysis of the three age groups using the chi-squared test yielded no significant differences in the distribution of impact levels between the age groups.

**Fig 5:**
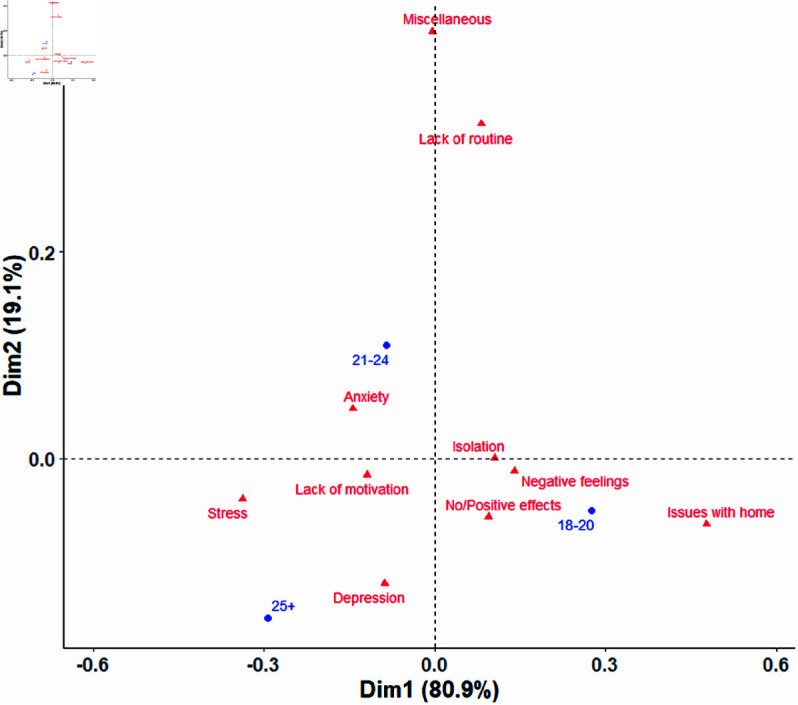
CA biplot illustrating the relationship between age groups (18-20, 21-24, 25+) and mental health factors. Dimension 1 (Dim1) explained 80.9% of the variance, while Dimension 2 (Dim2) accounted for 19.1%. The plot shows that the age group 25+ was closely associated with depression and stress. The 18-20 group aligned more with No/Positive effects, negative feelings, and issues with home. The age group 21-24 was linked to anxiety, lack of routine and miscellaneous factors.

Using CA, each age group shows distinct associations with specific mental health factors, illustrating how different age demographics experienced varied psychological impacts during the pandemic (see Fig 5). For the 25+ age group, the plot highlights stronger associations with depression and stress. The 18-20 age group aligns closely with factors like No/Positive effects, negative feelings, and issues with home, whereas for the 21-24 age group, the plot shows strong links to anxiety, lack of routine, and various stress-related factors.

## Discussion

This survey-based study was conducted in Spring 2020, at the beginning of the COVID-19 pandemic, and collected data related to college students’ mental health and academic success. Here we employ a combination of subgroup analysis and correspondence analysis to identify differences in how subsets of students divided across gender, major, degree type, age, and first generation status were impacted and explore the underlying reasons for these differences.

This study included data from well over 400 hundred students from a population of over 30,000 students. Some limitations of this study include the smaller sample size of males compared to females, and there lacked sufficient numbers to do a comprehensive analysis of differences among races. Also, the survey participants were self-selected to participate in the study, which could result in potential bias.

The classification of reasons provided by students was based on subjective interpretations provided by a subset of the research team, which may introduce potential errors or biases. Furthermore, as the survey was conducted at the onset of the pandemic without pre-pandemic data, it is not possible to determine whether the reported mental health issues predated the pandemic, were exacerbated by it, or were directly caused by it.

First-generation students were more closely linked with factors such as stress and lack of motivation. This may suggest that, during the pandemic, first-generation students may have been more prone to emotional challenges and motivational struggles, potentially due to the added pressures of navigating college without the support of an experienced family background in higher education. These results are in line with other survey studies across a larger population of college students that find first-generation students were more likely to report experiencing financial hardships, concerns about paying for college, unsafe living environments, food/housing insecurity and higher rates of mental health disorders than their non-first generation peers [23, 24].

We note that similar differences between these subgroups existed prior to the pandemic – in a multi-institutional study by Stebleton *et al*. [42]. They found that first-generation students tended to report greater levels of stress, along with lower levels of belonging and lower levels of use of services. Raposa *et al*. [25] found that first-generation students reported significantly less confidence in where to seek mental health support after the onset of the pandemic and their usage of mental health services dropped as well.

In contrast, non-first generation students were more strongly affected by factors such as anxiety, lack of routine, issues with home, and various miscellaneous challenges, including trauma, sleep disturbances, self-harm, and eating disorders. These findings indicate that, during COVID-19, non-first generation students may have been more affected by disruptions in their daily routines and the challenges of adapting to studying from home. In a pre-pandemic study, House *et al*. found that first-generation students reported more academic and financial stress, but had similar results when comparing mental health, and perceived family and social support [43]. It is possible that the pandemic exacerbated some of these factors.

For male students, there was a stronger association with No/Positive effects. They also appeared to have a more similar affect with females in regards to isolation and stress. This indicates that male students were more likely to report minimal impacts on their mental health, though some experienced isolation and stress. The higher association with No/Positive effects suggests that many male students may have perceived the pandemic’s effects as less disruptive. These results could also be due to the fact that there were fewer male students participating in the survey, and the ones who participated were more comfortable in participating due to the lack of impact.

Zhu *et al*. [44] found that student age, sex and whether the student was undergraduate versus graduate were not sources of variability in the rates of depression and anxiety symptoms of the pandemic. Instead they found that geographical location and the month in which the data was collected showed up as moderators. It is plausible that, since our study was performed at an university with a very diverse population (< 50% white students), that we were able to obtain different results.

In contrast to the male students, female students are more closely associated with a range of mental health factors, such as depression, lack of motivation, lack of routine, anxiety, miscellaneous factors, and issues with home. This broader set of associations suggests that female students encountered more significant challenges, with both emotional struggles and daily disruptions being more pronounced. The link to issues with home may indicate that household-related stress, such as family responsibilities or changes in living conditions, had a more substantial impact on their well-being during the pandemic. Many of these trends where female students were found with higher levels of depression and anxiety were also found in previously mentioned studies in the Introduction [16, 20, 21, 28].

In a global study at the beginning of the pandemic, Aristovnik, *et al*. found that males were less satisfied with the academic work and life compared to women. Females, on the other hand, were generally more affected by the pandemic in terms of their emotional life and personal circumstances [45]. Though the measurements are not identical, this study generally supports the results observed here. In another study with overlapping results involving Brazilian medical students, Pacheco *et al*. found that female students had a higher prevalence of depression, as well as mood and anxiety disorders. Males, on the other hand, were more associated with burnout [46]. Lui *et al*. also found an association of poorer mental health with female students [47].

While fewer studies examined outcomes for male students, Lee *et al*. [18] observed that men were more likely to identify beneficial opportunities during the pandemic, such as personal growth and time for reflection. However, the broader trend across studies highlights the greater emotional and psychological toll experienced by female students. These findings point to a clear gender disparity, with women facing amplified mental health challenges during the pandemic, which also aligns with the findings of our study.

We found that non-STEM students had stronger links to depression, stress, and anxiety. This pattern suggests that non-STEM students experienced a range of emotional and motivational challenges, possibly due to disruptions in their usual academic routines or struggles to stay engaged in remote learning environments. The association with depression and stress may indicate that the lack of structure and decreased social interactions had a more profound impact on non-STEM students, leading to higher levels of distress. The presence of miscellaneous factors, including eating disorders, substance abuse, and sleep issues, further suggests that non-STEM students faced a diverse set of serious stressors that contributed to these mental health difficulties.

On the other hand, STEM students show closer associations with isolation, negative feelings, issues with home, and No/Positive effects. The connection to isolation and negative feelings suggests that STEM students experienced social disconnection and emotional strain during the pandemic, possibly linked to the demands of their studies or the challenges of adapting to online learning. The association with issues related to home may indicate that household environments or responsibilities added to the stress experienced by STEM students. The link with No/Positive effects is likely due to the stronger presence of male students found in the STEM group than in the non-STEM subgroup.

While many studies examine the mental health impacts from the pandemic on STEM students (see, for example, [48–50]), few explore differences between STEM and non-STEM students. When comparing STEM and non-STEM majors, Sanchez-Pena, *et al*. observed higher depression and anxiety scores among engineering students compared to other groups including non-STEM and non-engineering STEM [51]. Cherikov *et al*. [14] found that, overall, STEM students surveyed during the pandemic screened positive for major depressive disorder and generalized anxiety disorder at a lower rate than student in humanities, arts, design communications and social/behavioral sciences. However, within STEM, students with majors in the physical sciences had more pronounced levels, screening positive at a rate comparable to some humanities and arts majors.

Undergraduate students more frequently reported factors such as issues with home, miscellaneous factors, negative feelings, and isolation. This suggests that undergraduate students were more likely to report challenges related to their living environments and social disconnection during the pandemic. The association with issues at home may reflect stress from household dynamics or disruptions in living situations, while the link to isolation indicates that being away from the campus setting and in-person interactions contributed to feelings of loneliness.

In contrast, graduate students were more closely associated with factors such as stress, depression, and lack of routine. Since graduate students are typically older than undergraduate students, many are likely to live on their own or possibly have families of their own, making issues with home less prevalent for graduate students compared to the undergraduates, which is reflected in this data. Also, since these students are generally older, they may have more responsibilities than undergraduates outside of school, which may lead to more reasons for stress, depression and issues with lack of routine.

In comparing mental health issues among undergraduate and graduate students, Wyatt and Oswalt [52] observed that undergraduate students exhibited higher rates of behaviors associated with poor mental health compared to graduate students, even before the COVID-19 pandemic. However, they also noted that graduate students reported higher levels of stress than their undergraduate counterparts. Similarly, Liu *et al*. [47] found that undergraduate students experienced poorer mental health outcomes than graduate students. Dial *et al*. [53] further reported that undergraduates demonstrated greater levels of stress, negative thinking, and lower positive mood than graduate students. During the pandemic, while mental health challenges increased for both groups, the rise was more pronounced among undergraduates. These findings, consistent with other studies, align with many, though not all of the results presented in this research.

For the 25+ age group, the analysis highlights strong associations with depression and stress, suggesting that older students may have faced heightened emotional and psychological challenges during the pandemic. Balancing advanced studies with work and possibly family obligations likely added to their stress levels. Research shows that adult learners, often juggling multiple responsibilities, are particularly vulnerable to burnout and depressive symptoms when routines are disrupted and stress levels increase [45, 54]. While non-traditional students, who fall within the 25+ age group, experienced higher increase in life stressors relative to their traditional peers during the pandemic, they also displayed higher levels of resilience according to Babb *et al*. [55]. The study of Raapar *et al*. [56] concludes that the support networks of non-traditional students tend to exclude formal university services and instead rely on family and fellow classmates. These students likely therefore had less disruption, at least in terms of support, than their traditional peers.

The 18–20 age group aligns closely with factors like No/Positive effects, negative feelings, and issues with home. These associations reflect some of the unique challenges faced by younger students, who may have struggled with the abrupt shifts in academic and social settings. Many in this age range depend heavily on peer connections, structured schedules, and campus living—all of which were disrupted during the pandemic. Studies suggest that younger students were more likely to report home-related issues, such as lack of personal space or increased family stress, contributing to negative feelings and isolation [11, 57]. However, the association with No/Positive effects hints that some younger students adapted well to online learning and may have appreciated the flexibility it provided, potentially finding new ways to manage their studies from home.

For the 21–24 age group, the plot shows strong links to anxiety, lack of routine, and various stress-related factors, indicating a more complex set of mental health challenges. This age group often includes upper-level undergraduates or early graduate students, who may have faced heightened academic pressures as they neared graduation or transitioned into advanced studies. The association with anxiety and isolation likely reflects concerns about academic and career uncertainties intensified by the pandemic [12]. Additionally, the connection to lack of routine and miscellaneous issues, such as sleep problems, trauma, or substance use, suggests that overlapping stressors affected their well-being. Students in transitional academic phases may be particularly vulnerable to stress and mental health challenges, which were further intensified by the pandemic’s destabilizing effects [20].

Due to the size of the data set, we were able to analyze the short answer questions individually without the use of artificial intelligence, as well as obtain reasonable conclusions. The open-endedness of the questions allowed a more robust investigation of the impact of the pandemic on the students. On the other hand, some subgroups of the population were not sufficiently present in the data in order to make any conclusions. This was an unique opportunity to examine how the pandemic as well as the protocols aimed at limiting its spread, affected the mental health of college students.

## Conclusion

Overall this broad study of college students’ mental health during the early stages of the COVID pandemic showed multiple results in the similarities and difference among subgroups of students. Many of the results were at least in part similar to studies that focused on a particular breakdown of college students, though there were some minor differences found. We demonstrated two different statistical analyses to arrive at our results given the challenging nature of the size (> 400) and type of survey data (i.e. numerical and text) collected. Subgroup analysis of the numerical ratings, based on self-reported demographic data, show differences in the level of impact from COVID by sex and first generation status are statistically significant. Differences in the level of impact across other demographic factors such as age, field of study and academic status (i.e. undergraduate vs. graduate) are not statistically significant. Correspondence analysis of the free response data provides insights into differences in how the subgroups were impacted, regardless of whether or not the level of impact is significantly different in the numerical ratings.
